# EMERGENCY DEPARTMENT USE AMONG OLDER ADULTS RECEIVING MEDICAID HOME- AND COMMUNITY-BASED SERVICES

**DOI:** 10.1093/geroni/igad104.1499

**Published:** 2023-12-21

**Authors:** Richard Fortinsky, Julie Robison, Dorothy Wakefield, James Grady, David Steffens, Deborah Migneault, Chae Man Lee

**Affiliations:** University of Connecticut, Farmington, Connecticut, United States; UConn Health, Center on Aging, Farmington, Connecticut, United States; University of Connecticut, Farmington, Connecticut, United States; University of Connecticut, Farmington, Connecticut, United States; University of Connecticut School of Medicine, Farmington, Connecticut, United States; UCONN Health, Farmington, Connecticut, United States; Univ of CT Health, Farmington, Connecticut, United States

## Abstract

Medicaid-funded home and community-based services (HCBS) for older adults aim to prolong independent living despite physical and cognitive disabilities. Medicaid-eligible HCBS populations are racially and ethnically heterogeneous, with multiple chronic conditions. Clinical threats to continued independent living among older adult HCBS recipients include acute illnesses, injuries, and exacerbations of chronic conditions, which could trigger a pathway of emergency department (ED) visits, hospitalization, and long-term nursing home admission. Despite its importance in the pathway from independent living, little is known about the rate of, or risk factors for, ED use in this target population. Accordingly, we determined predictors of ED use in a statewide cohort (N=5,651) in Connecticut’s Medicaid HCBS program for adults age >65, who were clinically assessed using a uniform tool between April 2020-March 2021. ED use, (0 vs 1+ visits), was ascertained over 12 months following clinical assessment using Medicare and Medicaid claims data. Predictor variables were racial and ethnic group, primary language, age, sex, educational attainment level, dementia diagnosis or moderate-severe cognitive impairment, depressive symptom severity, and Charlson comorbidity index. Cohort members were 76% female, 30% Hispanic, 15% non-Hispanic Black, 4% non-Hispanic Asian, 33% age >85. Forty-one percent had 1+ ED visits. Multivariate logistic regression analyses revealed that ED use was associated with (adjusted odds ratios p<0.05): high school education, more severe depressive symptoms, and more chronic conditions. Non-Hispanic Asian individuals had lowest ED visit likelihood. ED use among older adults receiving Medicaid HCBS might be reduced if depressive symptoms and multiple chronic conditions are optimally managed.

